# Laparoscopic Primary Colorrhaphy for Acute Iatrogenic Perforations during Colonoscopy

**DOI:** 10.1155/2013/823506

**Published:** 2013-02-07

**Authors:** Eric M. Haas, Rodrigo Pedraza, Madhu Ragupathi, Ali Mahmood, T. Bartley Pickron

**Affiliations:** ^1^Division of Minimally Invasive Colon and Rectal Surgery, Department of Surgery, The University of Texas Medical School at Houston, Houston, TX 77030, USA; ^2^Colorectal Surgical Associates, LLP, Ltd., Houston, TX, USA

## Abstract

*Purpose*. We present our experience with laparoscopic colorrhaphy as definitive surgical modality for the management of colonoscopic perforations. *Methods*. Over a 17-month period, we assessed the outcomes of consecutive patients presenting with acute colonoscopic perforations. Patient characteristics and perioperative parameters were tabulated. Postoperative outcomes were evaluated within 30 days following discharge. *Results*. Five female patients with a mean age of 71.4 ± 9.7 years (range: 58–83), mean BMI of 26.4 ± 3.4 kg/m^2^ (range: 21.3–30.9), and median ASA score of 2 (range: 2-3) presented with acute colonoscopic perforations. All perforations were successfully managed through laparoscopic colorrhaphy within 24 hours of development. The perforations were secondary to direct trauma (*n* = 3) or thermal injury (*n* = 2) and were localized to the sigmoid (*n* = 4) or cecum (*n* = 1). None of the patients required surgical resection, diversion, or conversion to an open procedure. No intra- or postoperative complications were encountered. The mean length of hospital stay was 3.8 ± 0.8 days (range: 3–5). There were no readmissions or reoperations. *Conclusion*. Acute colonoscopic perforations can be safely managed via laparoscopic primary repair without requiring resection or diversion. Early recognition and intervention are essential for successful outcomes.

## 1. Introduction

Iatrogenic perforation represents an uncommon yet potentially life-threatening complication during colonoscopy [1, 2]. Traditionally, patients have required open surgery with either primary repair of the perforation or bowel resection with or without ostomy creation [1, 3, 4]. Although these procedures are an effective approach, they often require large open incisions and may be associated with high complication rates, such as wound infection and hernias [1, 4]. In addition, the open approach usually results in slower recovery with longer hospital stay [5–7].

Minimally invasive colorectal surgery represents an efficacious alternative to the open approach, utilizing smaller incisions and resulting in diminished postoperative pain, earlier recovery, and lower postoperative morbidity [5–9]. Laparoscopic intervention has more recently been reported for the definitive treatment of acute colonoscopic perforations. This approach has shown to be a viable option, resulting in enhanced recovery in comparison to open primary colorrhaphy [5–7]. We began utilizing minimally invasive surgical (MIS) technique for repair of colonoscopic perforations in an effort to provide a safe and efficacious alternative to an open procedure. Our aim was to assess and report our initial experience with laparoscopic primary repair of acute colonic perforations during colonoscopy. 

## 2. Patients and Methods

Between October 2008 and March 2010, consecutive patients presenting with acute iatrogenic colonic perforation during colonoscopy were evaluated. Laparoscopic surgical repair of the perforations was performed by one of three board-certified colorectal surgeons (A. Mahmood, T. B. Pickron, and E. M. Hass) with extensive experience in minimally invasive procedures. Preoperative data including age, gender, body mass index (BMI), American Society of Anesthesiologists (ASA) score, indication for colonoscopy, and time interval between perforation and surgery were assessed. Intraoperative parameters including estimated blood loss (EBL), conversion to open surgery, and complications were collected. With respect to postoperative data, return of bowel function, resumption of oral intake, complications, length of stay (LOS), secondary interventions, and readmissions within 30 days after discharge were evaluated. 

### 2.1. Operative Technique

Initial entry into the peritoneal cavity was achieved under direct visualization using an Optiview trocar (Ethicon Endo-Surgery Inc., Cincinnati, OH, USA). An additional two or three 5 mm trocars were utilized with placement dependent on the suspected location of the perforation. Laparoscopic exploration was performed and followed by identification and isolation of the site of the colonic perforation. Any bowel spillage was aspirated, and the area was irrigated. The necrotic edges of the perforation were debrided, and colorrhaphy was performed with interrupted 3-0 Vicryl (Ethicon Inc., Somerville, NJ, USA) suture in a single layer technique. An air insufflation test was performed in all cases to confirm the integrity of the repair. 

## 3. Results

Five female patients presented with acute iatrogenic colonic perforation, which occurred during screening colonoscopy. The mean age, mean BMI, and median ASA of the patients were 71.4 ± 9.7 years (range: 58–83 years), 26.4 ± 3.4 kg/m^2^ (range: 21.3–30.9 kg/m^2^), and 2 (range: 2-3), respectively (Table 1). Three perforations were secondary to mechanical trauma and recognized during the colonoscopy, while two perforations occurred due to thermal injury and were identified within 24 hours of the colonoscopy. The perforations were located in the sigmoid (*n* = 4) and cecum (*n* = 1). While in 3 cases the time interval between perforation and surgery was 3-4 hours, in 2 cases surgery was performed following 18 and 20 hours of perforation. 

All procedures were successfully performed using pure laparoscopic technique. There was no significant blood loss (range: 0–50 mL) or intraoperative complications during the procedures, and none required conversion to open surgery. Surgical resection and diversion were not required for any of the perforations. Mean resumption of oral intake and return of bowel function, as evidenced by passage of flatus, were 1.4 ± 0.5 and 1.6 ± 0.9 days, respectively (range: 1-2 days). The average length of hospital stay (LOS) was 3.8 ± 0.8 days (range: 3–5 days). There were no postoperative complications, and none of the patients required readmission or secondary operative intervention (Table 2).

## 4. Discussion 

Although complications during colonoscopy are uncommon, colonic perforation represents a potentially life-threatening event that may result in peritonitis, sepsis, and multiorgan failure, thus demanding prompt diagnosis and intervention [1]. While colonic perforations have traditionally been managed through emergent laparotomy with segmental resection and possible diversion, MIS techniques, including laparoscopic segmental resection or primary suture repair and endoscopic suturing or clipping, have more recently been implemented [1, 3–7, 10–12]. The utilization of laparoscopic modalities has demonstrated to result in diminished surgical trauma, lower conversion rates, reduced complication rates, and quicker recovery with shorter length of hospital stay compared with open surgery [5–7].

Four main mechanisms have been hypothesized in the pathogenesis of colonoscopic perforation: direct penetration of the bowel wall, barotrauma, thermal abrasion, and traction injury [3, 13, 14]. The selection of an appropriate approach for the management of a colonoscopic perforation must be individualized on a case-by-case basis. A history of previous colonic pathology requiring partial colectomy, such as recurrent diverticulitis or neoplastic disease, may preclude consideration of primary repair. Lack of optimal bowel preparation prior to colonoscopy or a prolonged interval between perforation and intervention may increase the risk of fecal contamination of the peritoneal cavity. In such cases, resection with diversion may be considered [6, 13]. However, preservation of a minimally invasive platform may be accomplished through laparoscopic segmental resection [6]. Furthermore, some colonoscopic perforations may be managed with endoscopic clipping or with conservative measures [11, 15–17]. When identified during the index colonoscopy, endoscopic clipping may be successfully accomplished, avoiding any further intervention and its potential complications [11, 17]. Delayed colonoscopic perforations are typically due to thermal injury, which are in most cases small perforations. These minor perforations represent the main indication for conservative treatment, which consists of intravenous hydration, antibiotics, and bowel rest [16].

**Figure 1:**
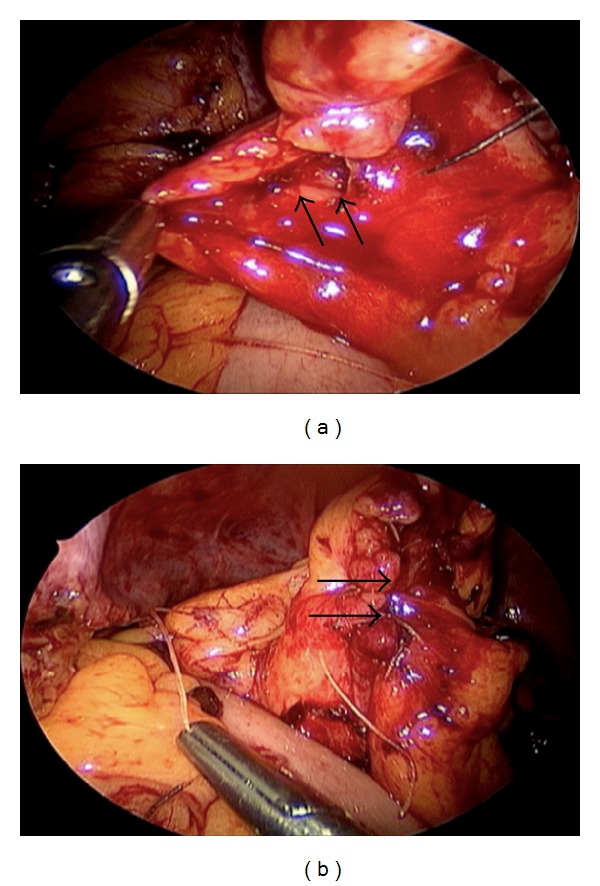
(a) Intraoperative image showing the colonic perforation (arrows) during laparoscopic exploration. (b) Intraoperative image showing the successful laparoscopic primary repair of the colonic perforation (arrows).

Laparoscopic surgery represents an efficient technique for primary colonic repair. During this MIS technique, laparoscopic exploration is performed to visualize the perforation and assess the bowel content spillage into the peritoneal cavity. It is important to examine the entire large bowel in order to identify and repair secondary perforations. Occasionally, the proper identification of the perforation is not readily achieved; in such cases, colonoscopic assistance may be required. In this scenario, colonoscopic insufflation with  CO_2_ is preferred over air insufflation, as the former is avidly absorbed through the colonic mucosa, avoiding substantial increment in the intraluminal pressure. Minimization of spillage is achieved by clamping the proximal bowel and using steep Trendelenburg for right colon perforations or reverse-Trendelenburg for left colon perforations. Once the colonic wall injury is identified, the edges of the perforation must be debrided if necrotic. This maneuver is challenging when performed laparoscopically, as the surrounding mesentery may be damaged resulting in considerable bleeding. Most colonic perforations occur in the antimesenteric bowel border; however, when the mesenteric bowel border is involved in the perforation, it must be sutured initially to avoid a residual unrepaired wall defect in the mesenteric commissure of the perforation. The colorrhaphy itself consists of interrupted stitches with absorbable suture, usually in one layer to avoid narrowing of the lumen, especially in the sigmoid, and to minimize stretching of the serosal layer (Figure 1). Prior to the completion of the procedure, an air insufflation test is recommended to evaluate the integrity of the repair. 

**Figure 2:**
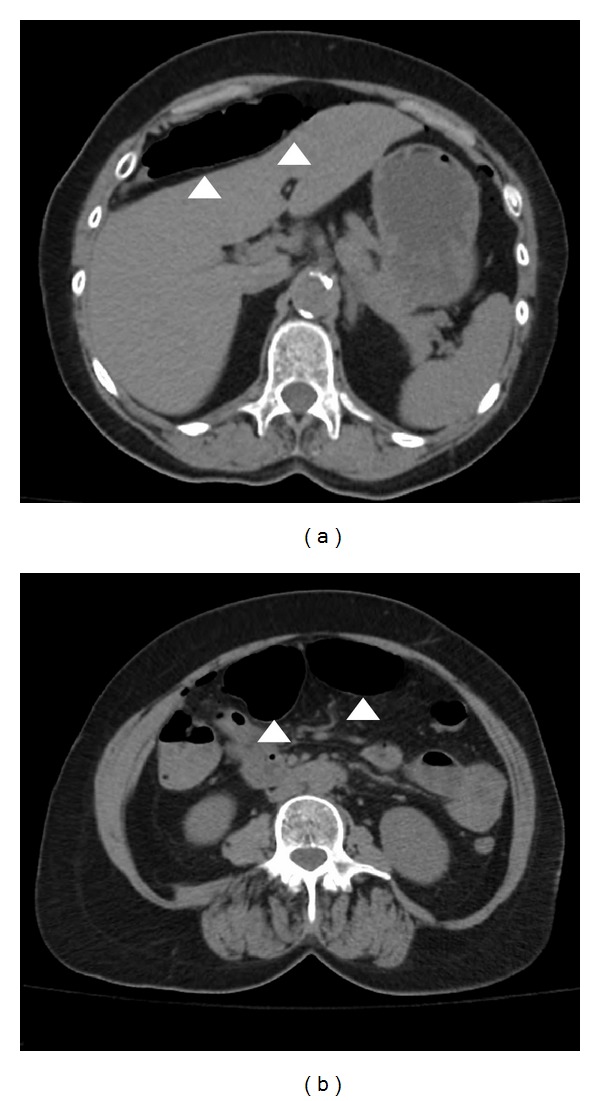
Abdominal CT scan images of a patient with colonoscopic perforation. The images show intraabdominal free air (arrowheads).

In our series, the majority of perforations (*n* = 3) were secondary to direct penetrating trauma from the tip or shaft of the endoscope. They were recognized during the colonoscopy, and the patients were taken to the operating room within 4 hours of occurrence. Laparoscopic exploration revealed absence of significant spillage or peritonitis and presence of viable tissue at the edges of the perforation. Primary colorrhaphy was successfully completed for management in all three cases. Two patients developed delayed perforation due to thermal injury. In the first case (patient 3), the perforation was secondary to polypectomy with argon-plasma coagulation, whereas in the second case (patient 5), the perforation occurred following ablation of 2 large cecal angiodysplasias. Both patients presented to the emergency department within 20 hours of their respective colonoscopies with mild generalized abdominal pain and absence of peritoneal signs on physical exam. Free air was noted on abdominal radiologic studies (Figure 2). Laparoscopic exploration revealed perforation with necrotic edges in both cases. Following debridement, laparoscopic primary colorrhaphy was successfully performed. 

Our postoperative outcomes compared favorably with those reported in the published literature. We encountered a mean length of hospitalization of 3.8 days, and there were no postoperative complications. In 2007, Hansen et al. [10] evaluated their experience with laparoscopic primary repair in 7 cases of colonic perforation. The overall mean LOS was 7.6 days, and they encountered two (28.6%) postoperative complications. One patient developed new onset atrial fibrillation, which resolved spontaneously. The remaining complication consisted of an intraabdominal abscess secondary to leakage at the site of the colorrhaphy, requiring sigmoid resection and end colostomy creation. In 2008, Rumstadt et al. [5] reported a case series that evaluated 13 cases of primary colon repair for free colonic perforations following colonoscopy. Laparoscopic approach was initially attempted in 10 patients; however, 2 cases required conversion to open technique due to severe peritonitis. The average LOS for the laparoscopic group was 7.1 days. This rather prolonged LOS was in part due to the discouragement of early discharge in the institutions in which the procedures were performed. Despite this, the authors reported significantly shorter LOS for the laparoscopic approach in comparison to the open technique (7.1  versus  14.3 days, *P* = 0.019). In the same year, Bleier et al. [6] published a study comparing outcomes following open and laparoscopic primary repair for the management of iatrogenic colonic perforations. Patient demographics were similar between both groups. The LOS was significantly shorter for the laparoscopic group (5  versus  9 days, *P* = 0.01). Furthermore, the complication rate was lower in the laparoscopic group (2/12  versus  5/7, resp., *P* = 0.01). In this comparative study, the authors concluded that the laparoscopic primary repair, when performed by experienced laparoscopic surgeons, is advantageous over the open technique. 

The present study evaluated the outcomes of our initial experience utilizing laparoscopic primary repair for the treatment of acute iatrogenic colonic perforations during colonoscopy. We found this minimally invasive approach to be safe and feasible for such cases. Accordingly, we currently consider this modality as an initial approach for the management of such perforations. If favorable conditions exist (e.g., minimal spillage, absence of sepsis), we could primarily repair. Otherwise, laparoscopic resection with ostomy creation should be entertained. None of our cases required conversion to open surgery; however, if the minimally invasive platform proves unsuccessful, a conversion to laparotomy can be readily performed.

## 5. Conclusion

Laparoscopic primary colorrhaphy is a safe and feasible approach for the management of acute colonoscopic perforations. Conventional laparoscopic suture repair facilitates a minimally invasive procedure with minimal surgical trauma, rapid postoperative recovery, and low complication rate. Early comparative studies have demonstrated comparable efficacy with open techniques for repair of perforations. Consequently, laparoscopic primary colon repair may increasingly play an important role as a therapeutic option in the future management of various perforations. Additional prospective comparative studies will be necessary to further elicit the benefits and limitations of this approach.

## Figures and Tables

**Table 1:** Preoperative and intraoperative parameters.

Patient	Gender	Age (years)	BMI (kg/m^2^)	ASA	Perforation site	Mechanism of perforation	Time between perforation and surgery (hours)	Conversion to open procedure	Intraoperative complications
1	F	67	26.3	2	Sigmoid	Direct trauma	4	No	No
2	F	83	21.3	3	Sigmoid	Direct trauma	3	No	No
3	F	58	30.9	2	Sigmoid	Thermal injury^¶^	18	No	No
4	F	78	26.1	3	Sigmoid	Direct trauma	3	No	No
5	F	71	27.5	2	Cecum	Thermal injury^¥^	20	No	No
Overall^§^	Female 100%	71.4 ± 9.7	26.4 ± 3.4	2	Sigmoid: 80%; Cecum: 20%	Direct trauma: 60%; Thermal injury: 40%	9.6 ± 9.3	0%	0%

ASA: American College of Anesthesiologists Score; BMI: Body Mass Index.

^§^Mean ± standard deviation, except ASA, which is represented as median.

^¶^Thermal injury following anterior rectosigmoid polypectomy.

^¥^Thermal injury following ablation of two incidentally found large cecal angiodysplasias.

**Table 2:** Postoperative outcomes.

Patient	Return of oral intake (days)	Bowel function recovery (days)	Length of Hospital stay (days)	Complications	Reoperation	Readmission
1	2	1	5	No	No	No
2	1	2	4	No	No	No
3	2	1	4	No	No	No
4	1	1	3	No	No	No
5	1	1	3	No	No	No
Overall^§^	1.4 ± 0.5	1.6 ± 0.9	3.8 ± 0.8	0%	0%	0%

^§^Mean ± standard deviation.
